# Optimizing equipment requirements and configuration rules for elderly home treatment environments: a rough set analysis framework

**DOI:** 10.3389/fmed.2025.1700646

**Published:** 2025-11-03

**Authors:** Rui Wang, Wei-Yan Ding, Zhan-Yan Zhou, Xiao-Cheng Li, Yi-Fan Zhang

**Affiliations:** ^1^College of Architecture and Design, Nanchang University, Nanchang, China; ^2^Institute for Research on Cultural Resources and Industries, Nanchang, China; ^3^Guangdong University of Finance and Economics, Guangzhou, China; ^4^College of Design and Innovation, Tongji University, Shanghai, China

**Keywords:** aging population, sub-health, home environment, home treatment devices, rehabilitation therapy, rough set analysis

## Abstract

**Background:**

With the acceleration of global population aging, elderly individuals in sub-health and pre-frailty states face increasing health risks that undermine quality of life. Home treatment devices offer a promising solution, but the key antecedent conditions and their configurational interactions remain unclear.

**Methods:**

This study adopted a configurational approach to identify and analyze the key antecedent conditions influencing elderly users' satisfaction. Potential indicators were collected through an expanded snowballing literature review and refined using the Fuzzy Delphi Method (FDM) with 18 experts (June–July 2025), resulting in nine key conditions across four dimensions. A structured questionnaire was administered to 163 elderly respondents (aged 60–80) in Qingdao, China. Rough set analysis (RSA) was applied, with key configuration selection based on a coverage threshold of 10%.

**Results:**

Ten valid rules were obtained, revealing multiple causal pathways for both satisfaction and dissatisfaction. The dominant satisfaction pathway (Safety = 5 and Cost Control = 5) covered 45.37% of cases, whereas the main dissatisfaction pathway (Safety = 2 and Usefulness = 2) covered 35.71%. High safety and usefulness are key conditions, but their effects depend on combinations with cost, cultural, health, and policy-related conditions. Dissatisfaction, in contrast, is often triggered by low levels of basic conditions. These findings demonstrate causal complexity, equifinality, and asymmetry, extending configurational theory to the domain of home treatment devices and providing practical guidance for device design and policy development.

## 1 Introduction

With rapid global population aging, delivering safe, effective, and operable care for older adults has become a salient medical challenge. By 2050, individuals aged 60 and above will account for 21.5% of the global population, reaching 2.1 billion (1). In China, by the end of 2023, there were 296.97 million people aged 60 and above (21.1% of the total population), including 216.76 million aged 65 and above (15.4%) (2). Institutional care is constrained by high costs, limited supply, and insufficient person-centered warmth (3), leaving diverse needs unmet; community-dwelling older adults are often overlooked (4), and economically disadvantaged groups require additional support (5). A sizeable proportion of elders live in a ‘sub-health' or pre-frailty state that, while below diagnostic thresholds, is linked to functional decline, lower quality of life, and accelerated chronic disease progression; without timely intervention, cardiopulmonary, musculoskeletal, and mental-health risks increase (6).

Against this backdrop, home treatment devices have emerged as an important complement to clinical care and rehabilitation. Devices such as portable oxygen concentrators, transcutaneous electrical nerve stimulation units, and infrared therapy instruments can provide continuous symptom management and functional support in home settings, enabling earlier intervention, better adherence, and greater continuity of care. For common conditions (e.g., chronic obstructive pulmonary disease, post-stroke disability, sarcopenia, or degenerative osteoarthritis), such devices show promising home-based applications (7–9).

However, effectiveness depends not only on the device itself but also on contextual enablers: spatial and cultural adaptation in the home, supportive community resources, and policy/reimbursement frameworks (10–12). In other words, device performance is co-determined by the fit between technology, user, and environment. Yet a systematic framework to evaluate this multidimensional adaptation, and to guide selection and application for sub-healthy elders, remains underdeveloped, motivating the present study.

Although existing research has provided valuable insights into the use of home-based therapeutic devices for older adults, it still lacks a systematic theoretical framework, particularly regarding the interactive mechanisms between multidimensional user needs and contextual factors. Some scholars have begun to recognize this complexity and introduced alternative methods to explore it. For example, applications of the KANO model have revealed asymmetric effects among factors, showing that the positive and negative impacts of the same attribute on satisfaction are not necessarily equivalent (13). DEMATEL analyses have further identified interrelationships among different factors, suggesting that the user experience of home-based treatment devices is inherently structural and systemic (14). However, while KANO is well suited for detecting asymmetrical effects and DEMATEL can examine causal relationships, neither method is capable of analyzing configurational effects, which represent a critical dimension of complexity. In recent years, fuzzy-set qualitative comparative analysis (fsQCA) has been used to uncover configurational mechanisms in elderly users' behavior and satisfaction, revealing multiple equifinal pathways and distinguishing core from peripheral conditions (15). While fsQCA is well regarded for addressing both configurational effects and causal asymmetry, its findings are sensitive to calibration choices and may deviate from the raw data structure, which can constrain how fully real-world complexity is captured. Despite accumulating insights, current research still lacks an integrated theoretical and methodological lens to systematically capture complex interactions. This gap calls for analytical approaches that transparently trace how user needs and contextual factors jointly produce outcomes in the use of home-based treatment devices for older adults.

To fill the above research gap, this study aims to reveal the antecedent conditions influencing elderly users' satisfaction with home treatment devices and their configurational relationships. Specifically, this study seeks to answer the following research questions:

- RQ1: What are the key antecedent conditions influencing elderly users' satisfaction with home treatment devices?
- RQ2: How do different configurations of these conditions jointly shape elderly users' satisfaction?

Empirically, this study focuses on elderly individuals in a sub-health state from a community in Qingdao. First, through a literature review and Fuzzy Delphi Method (FDM), potential antecedent conditions for home treatment devices are extracted. Next, using data from elderly individuals, rough set analysis (RSA) is employed to establish a configuration model and identify the causal logic linking these antecedent conditions to user satisfaction.

This study makes twofold contributions. Theoretically, it extends research on home healthcare by systematically examining the causal and configurational relationships between device conditions and user satisfaction, offering a nonparametric alternative to conventional approaches. Practically, the findings provide actionable insights for local governments and healthcare designers, helping to improve sub-health management, optimize device development, and promote sustainable home-based healthcare solutions.

## 2 Literature review

### 2.1 Potential conditions for home-based treatment devices

Most existing studies evaluate treatment devices from a single perspective (e.g., usability, technical performance, or clinical outcomes) without systematically integrating broader conditions which may influence user satisfaction. This narrow focus obscures the complexity behind elderly users' experiences. To address this gap, this study adopted an expanded literature search strategy. In addition to extracting candidate conditions from studies directly related to *elderly/older adults/aging* and *home-based/home care/home rehabilitation and treatment/assistive devices*, we also employed a snowballing approach (following references and citations) to identify additional factors that may influence user experience and satisfaction. These factors were ultimately organized into four analytical dimensions—device features, user needs, home and community environments, and policy orientations (see Table 1). The purpose of this inclusive approach was to ensure broad coverage of potential antecedent conditions. All preliminary indicators identified through this process were subsequently refined via the FDM and further distilled by RSA to determine the key antecedent conditions and their configurational relationships.

**Table 1:** Potential condition attributes.

**Dimension**	**Criteria**	**Description**	**References**
Device features dimension (*D*_1_)	Safety	Whether the use of the device can effectively protect older users' physical health and prevent accidental injuries	(14, 19, 21, 35)
	Usefulness	The impact on older adults' task execution time, workload, and cost	(20, 21, 35)
	Smart technology	The integration of artificial intelligence algorithms, information technology, the Internet, and dynamic adjustment services with health management	(14, 24, 27, 29, 70)
	Ease of use	Reducing operational steps so that older adults can independently use home-based treatment devices	(29, 69, 71)
User needs dimension (*D*_2_)	Health management needs	The functions, services, and support required to maintain and improve health	(27, 35, 72, 73)
	Affordability	The economic cost burden associated with using home treatment devices, including purchase, installation, and related expenses	(28, 74–77)
	Psychological resilience support	The support provided to help older adults cope with stress, challenges, and uncertainties in daily life	(24, 25, 78)
	Intergenerational digital inclusion	Older adults' ability to access, use, and benefit from digital technologies and devices on an equal basis	(30, 35, 79)
Environmental constraints dimension (*D*_3_)	Spatial adaptability	The degree to which the device is adaptable and operable within older adults' living environments	(34, 35, 80)
	Cultural adaptability	The extent to which the device aligns with older adults' values, lifestyles, behavioral patterns, and needs across different cultural contexts	(24, 33, 81)
Policy-oriented dimension (*D*_4_)	Cost control priority	The policies may include price control and quantity control, which aim to improve efficiency and address financial pressures within the healthcare system	(38, 77, 82)
	Universal policy requirements	Specific policy models designed to meet the diverse needs of older adults (e.g., relevant laws, pension insurance schemes, and user policy recommendations).	(23, 37, 77, 83)

#### 2.1.1 Device features

Device characteristics are key determinants of the successful adoption and use of home-based treatment devices by older adults (16). These devices have substantial potential to enhance older adults' quality of life and independence, positively affecting their health, safety, and comfort (17). However, older users often face operational, health management, and emotional challenges when using such devices at home (18), making device characteristics decisive for their acceptance and continued use.

Optimizing device characteristics to align with older adults' needs and abilities can significantly increase both acceptance and effectiveness. Existing studies on telemedicine services (19), smart devices (14), and assistive technologies (20) support a user-centered design approach. In practice, safety, smart technology, usefulness, and ease of use are critical for meeting older adults' needs. Safety is fundamental, as physical and cognitive decline with age heightens vulnerability; devices must therefore minimize safety risks (21). Older adults may also experience sensory and mobility impairments. In this context, ease of use concerns the intuitiveness of operation, while usefulness refers to whether the device effectively supports intended health outcomes (21). With technological advancement, smart technologies have become vital for improving older adults' quality of life (14). Their integration not only enhances functionality but also fosters understanding and trust through seamless interaction, better supporting health management and treatment needs.

#### 2.1.2 User needs

User needs critically shape product functionality, design decisions, and market acceptance. Conventional medical devices often feel cold and impersonal, neglecting older adults' specific needs. Beyond functionality and aesthetics, emotional connection plays a decisive role in adoption (22). Scholars have examined how features such as interactivity (23), privacy protection (24), and mindfulness design (25) influence users' psychological engagement with devices.

Older adults living alone often experience loneliness due to limited family interaction, while physical decline increases insecurity (26). Understanding their daily lives and psychological needs is therefore essential for enhancing device acceptance and effectiveness. Rising chronic disease prevalence further heightens attention to health management functions (27), while differences in economic capacity shape diverse preferences and priorities (28). Although older adults display varied attitudes toward digital technologies (29), improving digital inclusiveness can facilitate activity monitoring, reduce accidents, and enhance safety (30). At the same time, the effectiveness of these technologies depends on the users' psychological resilience and mental health (31). Ensuring that technological interventions do not generate psychological stress is equally important (32). In sum, older adults' needs are closely related to health management, affordability, psychological resilience, and intergenerational digital inclusion. These factors must be holistically addressed to ensure that devices improve both well-being and acceptance.

#### 2.1.3 Environmental constraints

Environmental constraints refer to contextual factors beyond the device itself that influence its use. Cultural perceptions may shape willingness to adopt (24) while compatibility with the home environment affects feasibility and user intention. Developers must therefore consider older adults' physical, social, and cultural contexts to ensure that devices meet their needs effectively (33).

Cultural orientation is particularly important. In autonomy-oriented cultures, older adults tend to cope independently with functional decline, whereas in interdependence-oriented cultures they may prefer family assistance (24). Moreover, user experience is shaped by multiple factors, including device-scenario compatibility, the role of spatial layout in interaction (34), and environmental adaptability (35). Consequently, spatial and cultural adaptability should be treated as important device characteristics to enhance both therapeutic outcomes and quality of life.

#### 2.1.4 Policy orientation

Public policy provides important guidance for addressing the needs of older adults (23). Structural, economic, and preventive policies can improve their quality of life and conditions across social, economic, and healthcare domains (36). Pension policies, in particular, play a central role in the broader elderly care system, shaping device development and adoption.

However, current policies remain insufficient (37). Legal frameworks, pension insurance, and user-oriented product policies require further refinement. Effective policy tools should address both supply- and demand-side considerations (38), including strategies to control costs or improve quality (21). These trends underscore the urgency of establishing a robust policy framework to address population aging. Consequently, policy orientation is considered a crucial analytical dimension alongside the others discussed above.

### 2.2 Complexity and configuration theory

Although prior studies provide empirical support for factors influencing home-based treatment devices, the relationships among these conditions are inherently complex—often nonlinear—and small changes can precipitate markedly different outcomes. For example, Zeng et al. (14) found that in elderly users' perceptions of technological products, perceived ease of use functions as a causal driver influencing other factors, whereas social dimension is more likely to be affected by other conditions. Moreover, the social dimension operates as a “must-be” condition: its absence causes a sharp drop in satisfaction, but its presence only leads to limited improvement. These findings highlight the intricate interactions and asymmetric relationships between different factors.

Most existing studies rely on traditional analytical methods such as Structural Equation Modeling (17, 39–41), which adopt linear and symmetric perspectives to explain user satisfaction and behavioral outcomes. While SEM is valuable for uncovering latent causal relationships, it is built on assumptions of stable, linear, and unidirectional associations among variables. These assumptions limit its ability to capture the dynamic interactions between diverse user needs and environmental factors in real-world contexts. Similarly, methods such as the Analytic Hierarchy Process (AHP) (21, 42) typically assume independence among variables, overlooking potential coupling, feedback loops, and contextual contingencies across different attributes. As a result, these approaches struggle to reveal the systemic and emergent structures underlying complex phenomena.

Configurational approaches offer a powerful alternative by conceptualizing complexity as clusters of interrelated conditions (43). Instead of isolating single variables, this perspective focuses on how different combinations of attributes jointly shape outcomes, enabling a more holistic understanding of causality (44). Configuration theory, based on the principle of causal asymmetry, posits that the conditions leading to a specific outcome may differ from those that fail to produce the same outcome (45). This perspective is particularly relevant in elderly home treatment contexts. For individuals in sub-health states, device selection and treatment effectiveness are rarely determined by a single factor; rather, they emerge from the interplay among multiple factors—such as ease of use and usefulness (15). Misalignment among these factors can lead to adverse outcomes, highlighting the importance of considering their joint and context-dependent effects. Configuration methods are well suited to address this challenge, as they can reveal how various factors interact, complement, or substitute for one another to produce different user experiences. While fsQCA is now commonly used to study configurational effects, RSA remains underused in this domain. Given its distribution-free, rule-based procedure that works naturally with ordinal (Likert) data, RSA is a promising tool for analyzing self-reported questionnaires on elderly care and rehabilitation products, yet it has been seldom applied in this specific context.

## 3 Research design and methods

**Figure 1:**
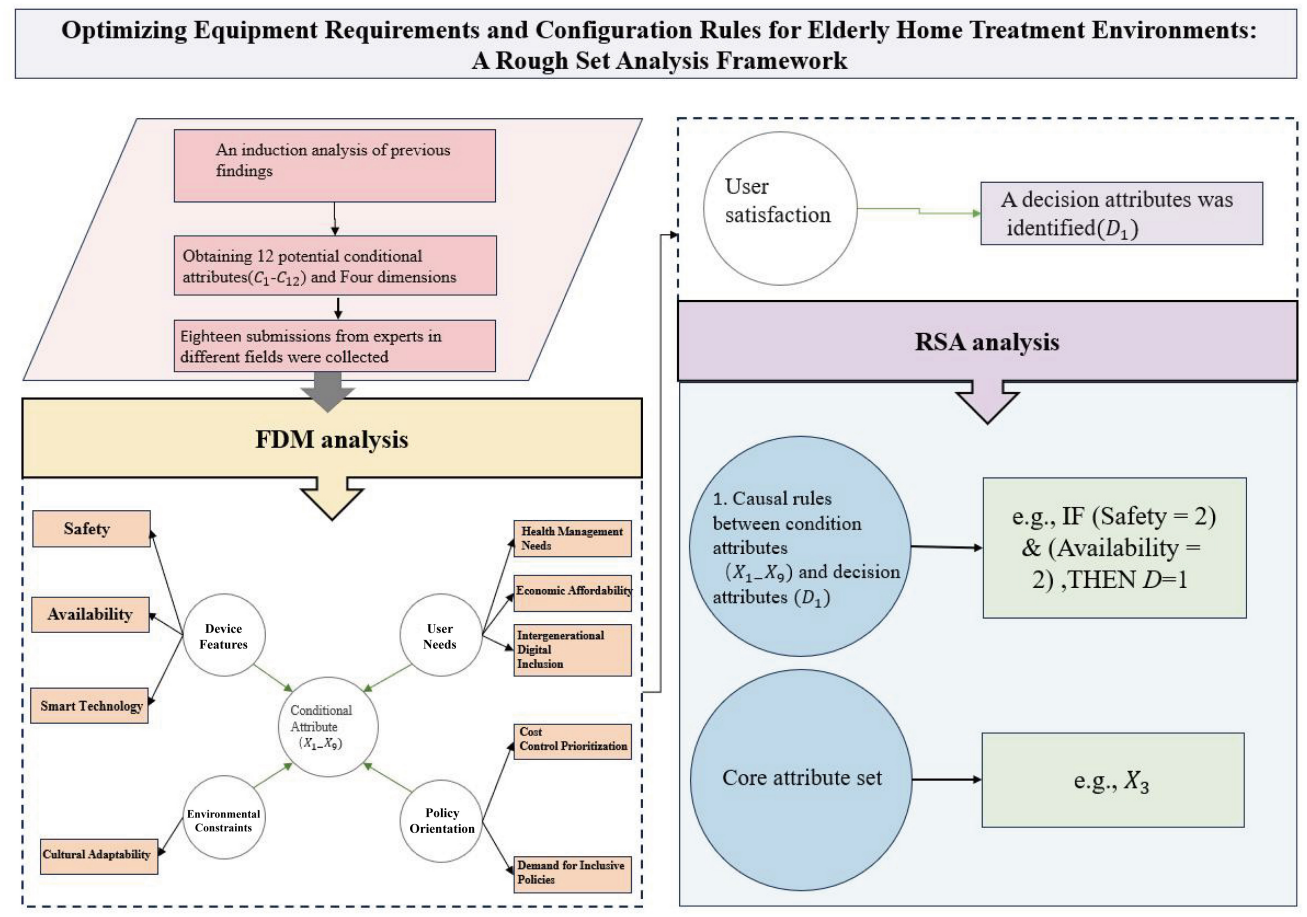
Research methodology.

The methodological framework of this study consisted of three stages (as shown in Figure 1).

In the first stage, we conducted a snowballing literature review to identify a broad range of potential antecedent conditions influencing elderly users' satisfaction with home treatment devices.

In the second stage, the FDM was applied with expert input to screen and refine these conditions, thereby developing a more systematic and reliable indicator framework.

In the third stage, RSA was employed to extract the key antecedent conditions and to analyze their configurational relationships and causal patterns.

This design ensures both the scientific validity of the indicator system and the robustness of the subsequent empirical analysis.

### 3.1 Fuzzy Delphi method

Given the complexity and diversity of the characteristics and functions of home treatment devices, quality assessment should reflect specific needs. Therefore, this study uses the FDM to evaluate the adaptability of various potential standards for home treatment devices.

The Delphi method, originally proposed by Dalkey et al. (46), is a structured approach designed to synthesize expert opinions and achieve consensus. Building on this, Ishikawa et al. (47) incorporated cumulative frequency distribution and fuzzy integral concepts, thereby developing the FDM. Jeng (48) further optimized FDM with a “gray area detection method” based on “double-triangular fuzzy numbers,” improving expert opinion convergence assessment.

Although the traditional Delphi method has been widely used, FDM offers significant advantages over the traditional method, particularly in addressing uncertainty and ambiguity issues (49). FDM integrates the opinions of all invited experts, not only saving time but also reducing the number of questionnaire distributions (50). Through fuzzy theory, FDM allows for a more reasonable and appropriate expression of experts' knowledge.

Following Jeng's (48) enhancement of the FDM, this study applies double-triangular fuzzy numbers and gray zone validation to assess the consistency and convergence of experts' understanding of home treatment devices. The implementation of FDM in this study proceeds as follows:

**Step 1: expert input**

Based on the established evaluation framework, an expert questionnaire is designed. Each expert provides an interval value for every evaluation criterion, specifying a *minimum value* *C*^*i*^ (the most conservative cognition) and *maximum value* *O*^*i*^ (the most optimistic cognition).

**Step 2: data processing**

For each criterion, all expert responses are collected. Outliers beyond two standard deviations are excluded. For the remaining data, the minimum, geometric mean, and maximum are computed, yielding triangular fuzzy numbers for both the *conservative cognition* $\left(C_{L}^{i},C_{M}^{i},C_{U}^{i}\right)$ and *optimistic cognition* $\left(O_{L}^{i},O_{M}^{i},O_{U}^{i}\right)$.

**Step 3: construction of triangular fuzzy numbers**

**Figure 2:**
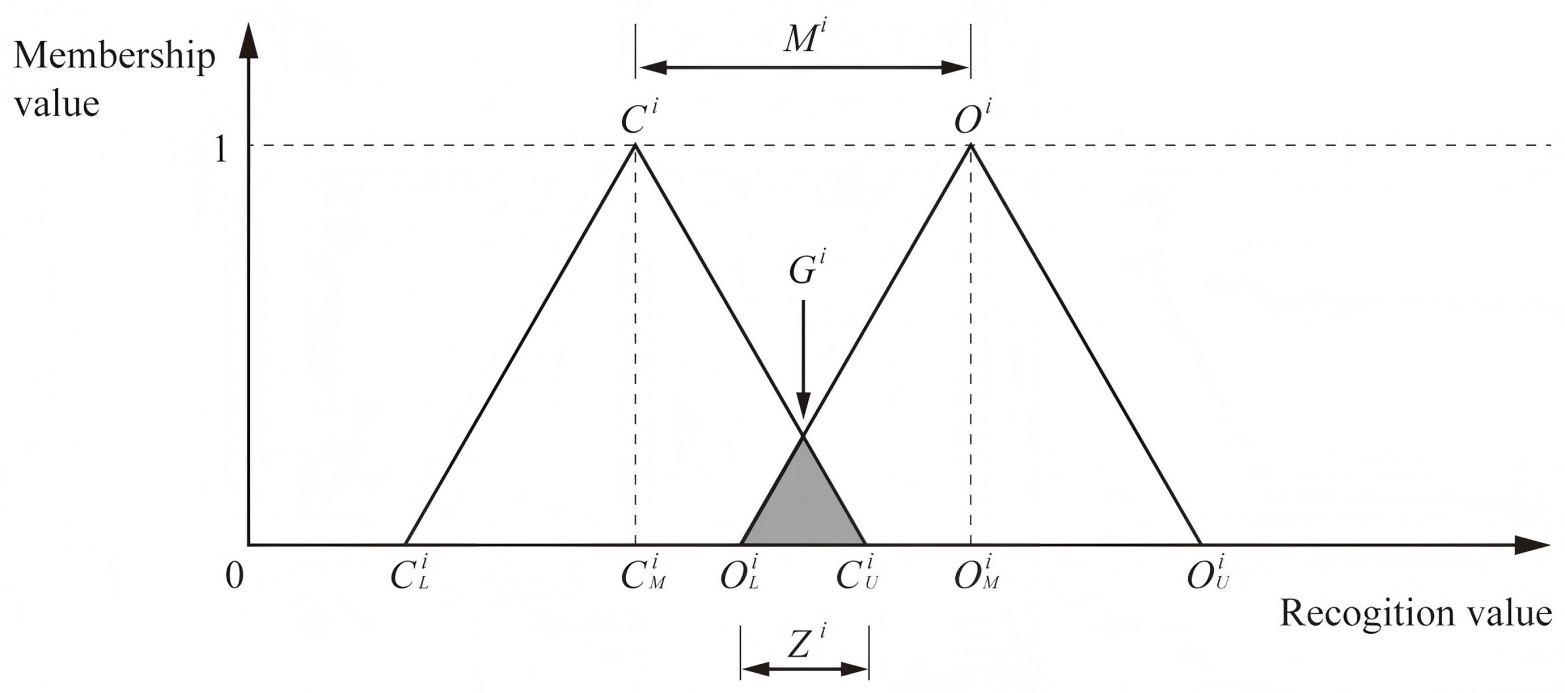
The triangular fuzzy numbers.

The triangular fuzzy numbers derived in Step 2 are used to represent the aggregated expert judgments (as shown in Figure 2).

**Step 4: convergence and consensus test**

The degree of overlap between the two triangular fuzzy numbers is examined to determine whether expert opinions have reached convergence:

If $C_{U}^{i}<O_{L}^{i}$, the two fuzzy numbers overlap completely, indicating convergence. The consensus value is calculated as the arithmetic mean of $C_{M}^{i}$ and $O_{M}^{i}$.

If $C_{U}^{i}>O_{L}^{i}$, partial overlap occurs. The gray zone ${Z^{i}=C}_{U}^{i}-O_{L}^{i}$ is compared with the interval between the two fuzzy numbers' midpoints. If within range, convergence is still considered valid; otherwise, divergence remains. In such cases, experts' opinions are re-examined and a second survey round is conducted until consensus is reached.

To determine whether a criterion should be retained, it is necessary to set a benchmark for consensus values. In this study, the benchmark was established at 7 out of 10, meaning that criteria with scores at or above this threshold were kept, while those below were excluded.

### 3.2 Rough set analysis

RSA, first introduced by Pawlak (51), provides a systematic approach for reducing attribute complexity and formulating decision rules. Its core principle is to approximate uncertain or imprecise concepts through the construction of lower and upper approximations based on equivalence relations. These approximations delineate the boundary of classification, enabling the identification of indispensable attributes, the elimination of redundancy, and the derivation of interpretable ‘if-then' rules.

For home treatment devices, this approach is particularly valuable because user satisfaction and therapeutic effectiveness are jointly influenced by diverse factors, including device functions, patient needs, environmental constraints, and policy requirements. By uncovering latent patterns in heterogeneous datasets, RSA reveals how different combinations of attributes shape outcomes, echoing holistic perspectives in design evaluation (52, 53). It also accommodates both favorable and unfavorable results, thereby exposing asymmetries in user decision-making that traditional parametric models may fail to capture (54). Methodologically, RSA is highly flexible. It relies on set-theoretic reasoning rather than assumptions of data distribution such as normality or independence (55). Moreover, its capacity to integrate quantitative performance indicators with qualitative user judgments within a unified framework enhances both its adaptability and explanatory power (56).

As a case-driven technique, RSA excels in complex decision-making contexts. Applied to home healthcare technologies, it reduces large and diverse datasets into a concise set of key determinants. The resulting decision rules highlight how particular configurations of device features influence patient acceptance and therapeutic outcomes. In doing so, RSA not only clarifies the causal mechanisms underlying device use but also provides an interpretable, evidence-based foundation for the optimization and design of home treatment devices.

The specific steps of the RSA outlined by Wang et al. (57), which include the following 6 steps:

**Step 1: construct the information system**

The relationship of the information system is defined as Equation R1. Here, *U* is a finite non-empty object set, *A* is a finite non-empty attribute set, and *A* can be divided into two finite non-empty conditional attribute sets *C* and decision attribute sets *D*. *V* is the set of attribute values, and *f* is the information description function.


(R1) $$\begin{array}{l} S\;=\;\left(U,\;A=C\cup D,\;V,\;f\right) \end{array}$$

**Step 2: establish indistinguishable relationships**

Let *B*⊆*C* be a subset of conditional attributes. An indistinguishable relation *IND*(*B*) defines which object pairs in the universal set *U* are indistinguishable under the attribute set *B*, as shown in Equation R2.


(R2) $$\begin{array}{l} IND\left(B\right)=\left\{\left(x,y\right)\in U|\forall a\in B,f_{a}\left(x\right)=f_{a}\left(y\right)\right\} \end{array}$$

This relationship divides the entire set *U* into equivalence classes*U*/*IND*(*B*) = {*I*_*B*_∣*x*∈*U*}, where each class contains all objects that are indistinguishable from *B* under the attribute set *x*. These equivalence classes serve as basic information units in rough set analysis and are crucial for constructing approximations and extracting decision rules in subsequent steps.

**Step 3: define upper approximation and lower approximation**

Given a target object *X*⊆*U*, it is possible to approximate *IND*(*B*) using equivalence classes derived from *X*. The lower approximation of *B**X* contains those objects that can be completely determined to belong to the target concept *X*, as shown in Equation R3. All equivalence classes of these objects are completely contained in *X*, representing precise knowledge.


(R3) $$\begin{array}{l} BX=\left\{x\in U|I_{B}\left[x\right]\subseteq X\right\} \end{array}$$

These approximations lay the foundation for calculating dependencies and extracting decision rules in subsequent steps.

**Step 4: confirm the dependency relationship of conditional attributes**

Confirm the condition attribute dependency formula as shown in Equation R4. Divide the object set *D* based on the indistinguishable relationship of the decision attribute *U*.


(R4) $$\begin{array}{l} U/IND\left(D\right)=\left\{D_{1},D_{2},\ldots ,D_{k}\right\} \end{array}$$

The dependency relationship between the conditional attribute *B* and the decision attribute *D*, as shown in Equation R5. The lower approximation *D* of the decision attribute is determined by the conditional attribute *B*, | |representing the number of objects. | *U* | represents the number of *U*. γ_*B*_(*D*) represents the dependency relationship between the decision attribute and the conditional attribute, with a range between 0 and 1. The closer to 1, the more dependent the decision attribute is on the conditional attribute.


(R5) $$\begin{array}{l} \gamma_{B}\left(D\right)=\frac{\left|BD\right|}{\left|U\right|} \end{array}$$

**Step 5: derive the reduction set and core attribute set**

As shown in Equation R6, RED (*Q*) is the reduction set of *C*, and RED (*Q*) contains or is equal to *C*.


(R6) $$\begin{array}{l} \text{RED}\left(\text{Q}\right)\subseteq \text{C} \end{array}$$

As shown in Equation R7. The intersection of all simplified sets is the core attribute set.


(R7) $$\begin{array}{l} \text{COR}\left(\text{C}\right)=\cap \text{RED}\left(\text{Q}\right) \end{array}$$

**Step 6: derive knowledge rules**

The reduced set of conditional attributes maintains the relevance between conditional attributes and the decision layer. Once the core attribute set is obtained, it can be used to generate decision rules that reflect the relationship between conditional attributes and decision outcomes. These rules retain the decision-making capabilities of the original dataset while using fewer attributes. Each decision rule can be expressed using Equation R8, where Φ and ψ represent the condition and decision of the rule, respectively. Rules are “if...then...” statements associated with condition classes and decision classes. Decision rules reflect the relationship between a set of conditions and decisions.


(R8) $$\begin{array}{r} \text{A decision rule in S is expressed as }\Phi \;\to \;\psi ,\\ \text{and read as if }\Phi \text{ then},\;\psi \end{array}$$

Support is expressed by Equation R9, and coverage ratio is defined by Equation R10. Support measures the proportion of samples in the data set that simultaneously satisfy the rule's premise and conclusion. If the support is high, it means that the rule applies to most of the data. Coverage measures the proportion of samples that satisfy premise Φ among all samples that satisfy conclusion ψ. The higher the coverage, the more meaningful the premise of the rule is in generating that conclusion.

sup_*S*_(Φ, ψ) represents the support of rule Φ → ψ. The number of samples in the dataset that simultaneously satisfy both prerequisite Φ and conclusion ψ.

card(∥ψ_∥_*S*_)−_ represents the number of samples in dataset *S* that satisfy premise ψ and conclusion ψ.

cov_*s*_(Φ, ψ) indicates the coverage of rule Φ → ψ. That is, among the samples in the dataset that satisfy conclusion ψ, how many samples also satisfy premise Φ.

card(∥ψ_∥_*S*_)−_ represents the number of samples in dataset *S* that satisfy conclusion ψ.


(R9) $$\begin{array}{lll} {\text{sup}}_{S}\left(\Phi ,\psi \right) & = & \text{card}{\left(∥\Phi \wedge \psi {∥}_{S}\right)}_{-} \end{array}$$

(R10) $$\begin{array}{lll} {\text{cov}}_{s}\left(\Phi ,\psi \right) & = & {\text{sup}}_{S}\left(\Phi ,\psi \right)/\text{card}{\left(∥\psi {∥}_{S}\right)}_{-} \end{array}$$

## 4 Data collection and analysis

### 4.1 Empirical case

According to the World Health Organization (WHO), individuals aged 65 and above are defined as elderly. When the elderly population in a country accounts for 7%, 14%, and 20% of the total population, the country is referred to as an aging society, a deeply aging society, and a super-aged society, respectively (58). Under economic pressure, the challenges posed by population aging have become increasingly prominent, especially in some district-level areas, where related issues and shortcomings are becoming more evident.

According to survey data from Shibei District in Qingdao, by the end of 2022, the number of residents aged 60 and above in Qingdao had reached 268,000, marking the city's entry into the aging phase. The issue of population aging is particularly pronounced in Shibei District, where the elderly population is predominant. In response to this challenge, the Qingdao municipal government has actively implemented national policies and proposed a series of solutions such as piloting the long-term care insurance scheme and expanding community-based elder care services (59). These initiatives not only demonstrate Qingdao's proactive approach to population aging but also make it a suitable setting for examining elderly users' demand for home treatment devices. Accordingly, this study takes Shibei District in Qingdao as a case, evaluating the demand for treatment devices among elderly individuals in a sub-health state within the home environment, and provides corresponding policy recommendations based on the research findings.

### 4.2 Revision of the evaluation framework

This study was conducted in China, and the questionnaire was translated into Chinese prior to the survey to facilitate the collection of expert opinions. An expert survey was carried out using the FDM questionnaire between June 7 and July 7, 2025. After receiving background information and survey instructions, participants first completed their personal information and then identified four dimensions and selected nine key attributes based on their professional judgment. In addition, all experts were invited to provide written comments and suggestions for each item.

All questionnaire items were rated on a ten-point Likert scale, ranging from 1 (completely unimportant) to 10 (extremely important), and experts were asked to provide both “conservative” and “optimistic” scores for each attribute.

To ensure the reliability of the sample, experts were selected using concrete eligibility criteria: (a) at least a master's degree; (b) a minimum of 3 years of relevant research or professional experience; (c) domain expertise in at least one of the following: elderly care, treatment/assistive device design and management, or community health services; and (d) at least one additional qualifier, such as participation in related research or policy projects, peer-reviewed publications, or a professional role tied to elderly care or rehabilitation devices. We distributed 20 expert questionnaires and received all back; 18 were valid and two were excluded for incompleteness, which aligns with prior FDM practice where panels of roughly 10–20 experts are commonly deemed adequate (60–62). Specifically, 18 experts held a master's or doctoral degree, and nine had more than 3 years of relevant research or work experience. The sample included six experts with medical backgrounds, three community managers with relevant field experience, and 10 experts from product management and research fields, including three specializing in geriatric sociology. Therefore, all experts had sufficient qualifications to participate in the survey.

Finally, the FDM screening yielded nine key antecedent conditions for the subsequent configurational analysis, thereby addressing RQ1 regarding the key conditions of home treatment devices. The FDM results are reported in Table 2.

**Table 2:** Results of FDM.

**Criteria**	***C_*U*_***	***O_*L*_***	***C_*M*_***	***O_*M*_***	***G^*i*^***	**Achieved the threshold?**	**Conditional attributes**
Safety	8	6	6.0666	8.4	7.1077	Yes	*X*_1_
Usefulness	9	7	6.9333	9.1333	8.0159	Yes	*X*_2_
Smart technology	8	7	6.7333	8.9333	7.6042	Yes	*X*_3_
Ease of use	7	5	4.4666	7.4667	5.9867	No	—
Health management needs	8	7	6	8.4	7.4118	Yes	*X*_4_
Economic affordability	8	7	5.5333	8.5333	7.3833	Yes	*X*_5_
Psychological resilience support	7	6	5.6667	8.0667	6.6078	No	—
Intergenerational digital inclusion	8	7	6	8.3333	7.4	Yes	*X*_6_
Spatial adaptability	7	6	5.7333	8.2	6.6346	No	—
Cultural adaptability	7	7	5.6667	7.9333	7	Yes	*X*_7_
Cost control prioritization	8	6	5.8667	8.3333	7.0448	Yes	*X*_8_
Demand for inclusive policies	8	7	5.9333	8.5333	7.4259	Yes	*X*_9_

### 4.3 Decision rule extraction

The survey for this phase was administered online via the Wenjuanxing platform (https://www.wjx.cn). The questionnaire consists of three parts. The first part collects background information from respondents, including gender, age, and product usage frequency. The second part asks respondents to recall their most recent experience using treatment devices, guided by a series of questions such as “Please provide the name of the treatment device you used most recently.” The third part focuses on the elderly population in Shibei District, Qingdao. Respondents are asked to evaluate treatment devices that left a strong impression on them, whether the experience was positive or negative. In this section, respondents assess each conditional attribute and decision attribute. Decision attributes (***D***) are evaluated using a semantic scale ranging from “dissatisfied (1)” to “satisfied (3)”; conditional attributes (***C***), which cover four dimensions and nine attributes extracted from the FDM results, are measured using a 5-point Likert scale ranging from “strongly disagree (1)” to “strongly agree (5)” (as shown in Table 3). The questionnaire data were collected from the elderly population in the old communities of Shibei District, Qingdao, between June and July 2025. A total of 226 questionnaires were collected, and after excluding invalid responses, 163 valid questionnaires were obtained, primarily due to the completeness of the questionnaire content. As shown in Table 4, the respondents were mostly aged between 60 and 80 years, with 77.3% of them using treatment devices at least three times per month, and most respondents had extensive experience using treatment devices.

**Table 3:** Description of the attributes in the primary survey.

**Attributes**	**Domain values**	**Value set**
Safety	Very dissatisfied, dissatisfied, neutral, satisfied, very satisfied	{1, 2, 3, 4, 5}
Usefulness	Very dissatisfied, dissatisfied, neutral, satisfied, very satisfied	{1, 2, 3, 4, 5}
Smart technology	Very dissatisfied, dissatisfied, neutral, satisfied, very satisfied	{1, 2, 3, 4, 5}
Health management needs	Very dissatisfied, dissatisfied, neutral, satisfied, very satisfied	{1, 2, 3, 4, 5}
Economic affordability	Very dissatisfied, dissatisfied, neutral, satisfied, very satisfied	{1, 2, 3, 4, 5}
Intergenerational digital inclusion	Very dissatisfied, dissatisfied, neutral, satisfied, very satisfied	{1, 2, 3, 4, 5}
Cultural adaptability	Very dissatisfied, dissatisfied, neutral, satisfied, very satisfied	{1, 2, 3, 4, 5}
Cost control prioritization	Very dissatisfied, dissatisfied, neutral, satisfied, very satisfied	{1, 2, 3, 4, 5}
Demand for inclusive policies	Very dissatisfied, dissatisfied, neutral, satisfied, very satisfied	{1, 2, 3, 4, 5}
**Overall**		
Satisfaction level	Dissatisfied, neutral, satisfied	{1, 2, 3}

**Table 4:** Characteristics of respondents.

**Variables**	***N* = 163**	**Percentage (%)**
**Gender**		
Male	85	52.14
Female	78	47.86
**Age**		
60–65	59	36.19
65 and above	104	63.81
**Living arrangements**		
Living in a nursing home	5	3.07
Living at children's homes	12	7.36
Living in one's own home	146	89.57
**Whether you live alone**		
Yes	24	14.72
No	139	85.28
**Whether to pay attention to the pension policy**		
Yes	129	79.14
No	34	20.86
**The number of times wellness equipment is used each month**		
Less than 3 times	37	22.7
More than 3 times	126	77.3
**Your health status**		
Self-care	156	95.71
Semi-self-care	6	3.68
Dependent on guardian	1	0.61
**Your monthly income status**		
≤ 3,000 yuan	62	38.04
3,000–6,000 yuan	44	26.99
≥6,000 yuan	57	34.97
**Was there any assistance from others in filling out this**		
**questionnaire?**		
Yes	136	83.44
No	27	16.56

The data from the third section were used for RSA analysis. We used the ROSE2 software developed by the Intelligent Decision Support Systems Laboratory at Poznań University of Technology to generate decision rules. In RSA, the accuracy and quality of approximations are key parameters for extracting decision rules. The overall classification quality was 1, and the approximation accuracy for the three decision classes are shown in Table 5, indicating that the attributes in the dataset can effectively classify users' demand levels. Smart technology is a core conditional attribute, meaning that it consistently plays a pivotal role and has a stable, significant impact on outcomes. The RSA decision rule extraction process can generate a large number of rules, which are crucial for the analysis as they help determine whether all rules played a significant role in the classification process. This study selected rules with a coverage rate of no less than 10% from the RSA results. Coverage rate is determined by calculating the ratio of the number of samples supporting the rule to the total number of samples in the corresponding decision class. Since this study aims to identify directions for improving medical treatment devices, only rules with “*D* = 1” and “*D* = 3” were considered, resulting in 10 final rules, with rules 1–5 corresponding to *D*_1_ and rules 6–10 corresponding to *D*_3_, as shown in Table 6. These rules specify how distinct combinations of antecedent conditions coalesce into negative and positive pathways, thereby addressing RQ2 on how configurations jointly shape elderly users' satisfaction. To ensure the accuracy and reliability of rule identification, we employed a 10-fold cross-validation method, and the final average accuracy was 71.07%, indicating that the data are suitable for RSA analysis, and the generated decision rules are reasonable and reliable.

**Table 5:** Accuracy and quality of classification.

**Class**	**No. of objects**	**Lower approximation**	**Upper approximation**	**Accuracy of classification**	**Quality of classification**
*D* = 1	14	14	14	1.0000	1.0000
*D* = 2	41	41	41	1.0000	
*D* = 3	108	108	108	1.0000	
Core attributes	*X*_3_				

**Table 6:** Casual rules.

**No**.	**Conditions (*C*)**	**Decision (*D*)**	**Proportion of training data covered by the rule (%)**
1	(Safety = 2) and (usefulness = 2)	Dissatisfied (*D* = 1)	35.71
2	(Economic affordability = 3) and (cost control prioritization = 2)		35.71
3	(Smart technology = 1) and (economic affordability = 1)		14.29
4	(Safety = 1) and (smart technology = 1)		14.29
5	(Usefulness = 1) and (health management needs = 1)		14.29
6	(Safety = 5) and (cost control prioritization = 5)	Satisfied (*D* = 3)	45.37
7	(Safety = 5) and (intergenerational digital inclusion = 3) and (demand for inclusive policies = 5)		11.11
8	(Usefulness = 4) and (cultural adaptability = 5)		17.59
9	(Usefulness = 4) and (health management needs = 4)		20.37
10	(Intergenerational digital inclusion = 5) and (demand for inclusive policies = 3)		13.89

## 5 Discussion

### 5.1 Theoretical implications

**Figure 3:**
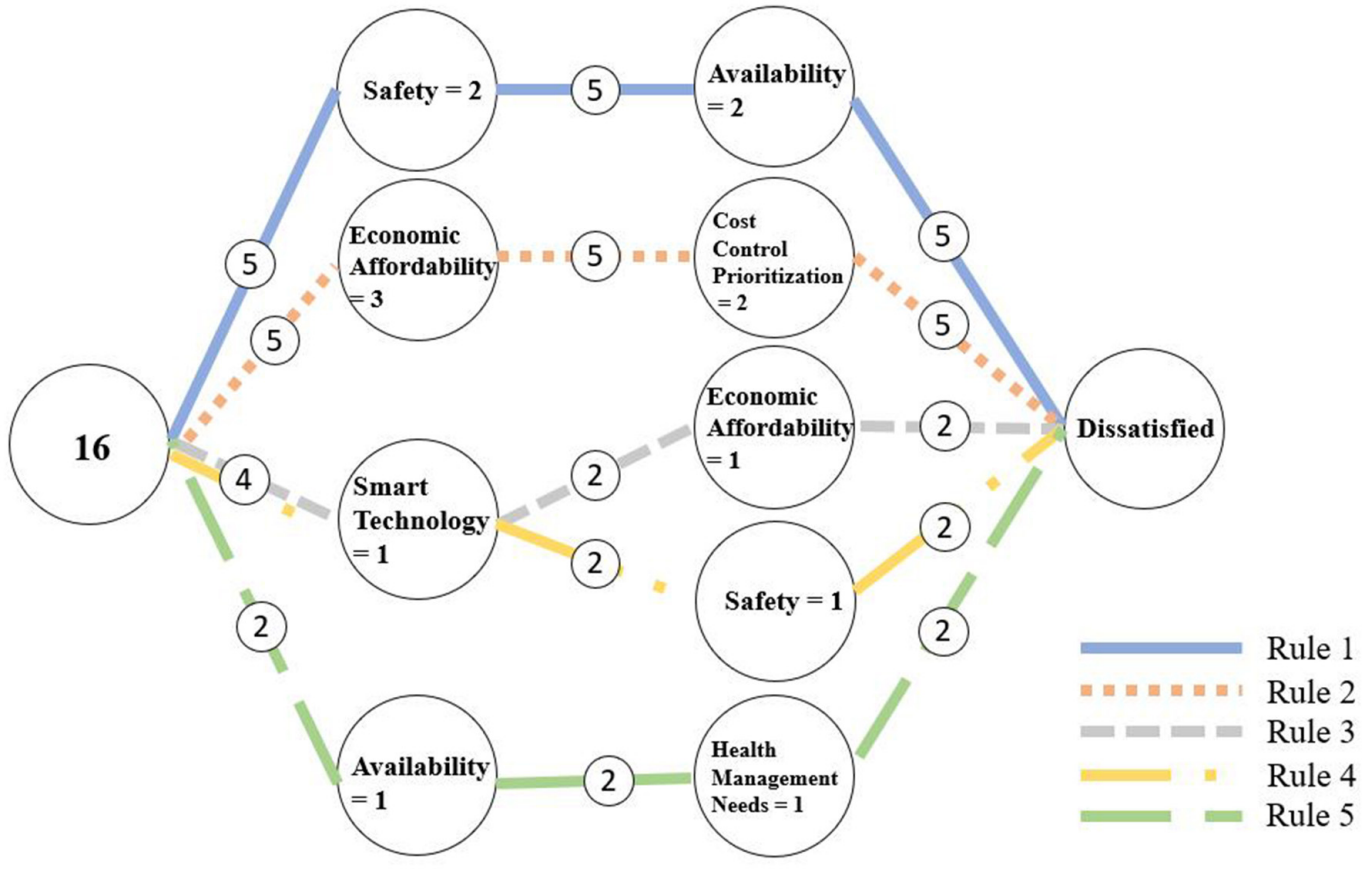
Rules of dissatisfaction.

**Figure 4:**
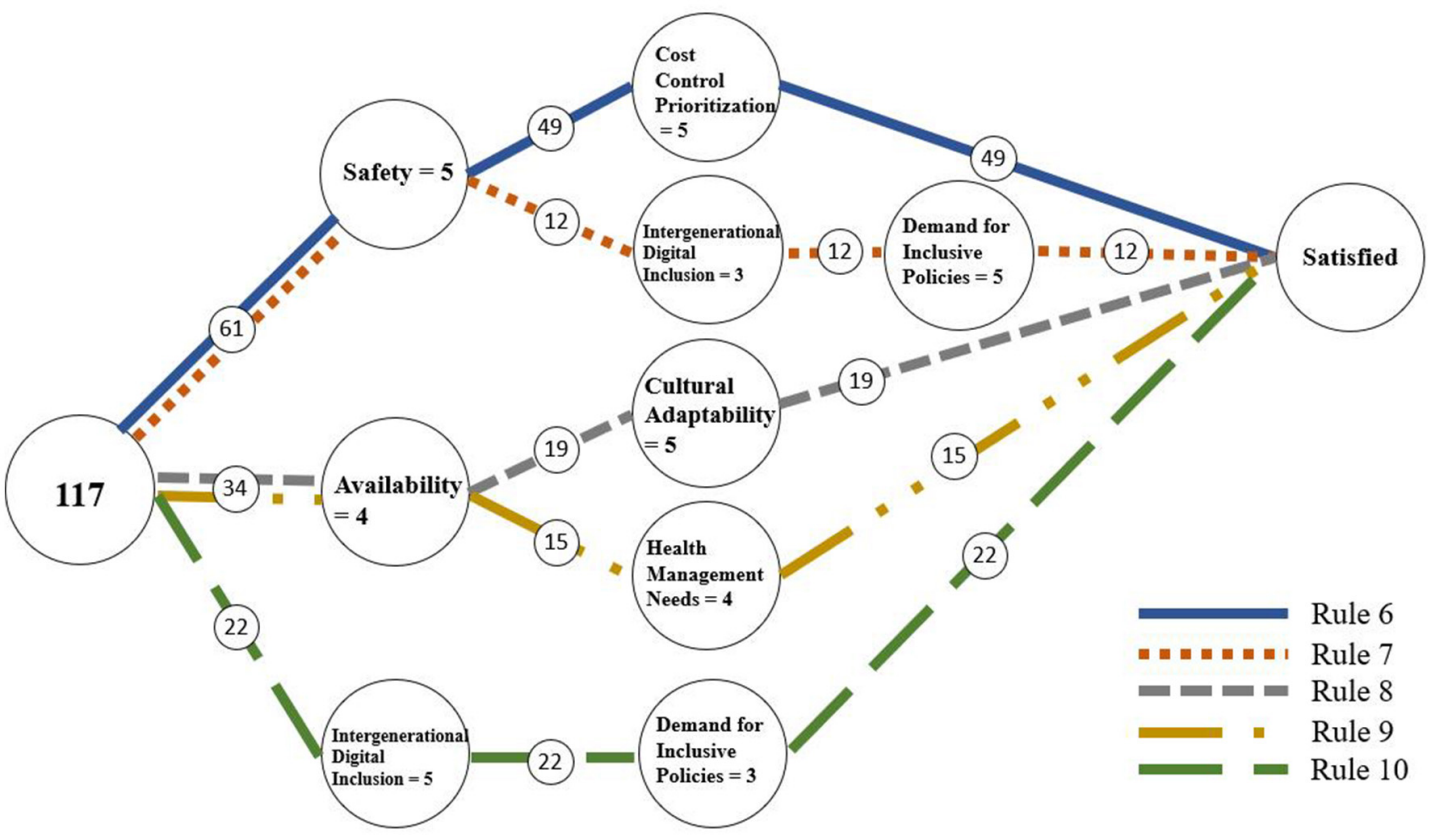
Rules of satisfaction.

From the perspective of configurational theory, these ten rules collectively reveal the causal complexity and multiple pathways underlying satisfaction and dissatisfaction, demonstrating clear configurational equifinality and pathway diversity. Overall, dissatisfaction (Rules 1–5) is generated by various combinations of low levels of key conditions, whereas satisfaction (Rules 6–10) emerges from different combinations of multiple high-level conditions. This indicates that satisfaction and dissatisfaction are not simply opposite ends of a single continuum but are driven by distinct causal structures. To further illustrate the conditional combinations and decision pathways, the study visualized these rules using a decision flow graph (56) (as shown in Figures 3, 4), which helps reveal the structural relationships and pathway differentiation among different configurations.

Beyond the insights offered by methods such as KANO, DEMATEL, and fsQCA, the configurational analysis in this study makes several distinctive theoretical contributions. KANO and DEMATEL have advanced understanding by identifying asymmetric effects and inter-factor influences but typically examine factors in isolation or through pairwise relations (14). Likewise, fsQCA uncovers multiple causal pathways, yet most applications focus on a narrow set of conditions and rarely integrate user needs, product attributes, environmental constraints, and policy contexts within a single analytical framework (15). In contrast, this study first established a multidimensional framework through literature review and FDM, then used RSA with user data to reveal how these antecedents interact to form satisfaction and dissatisfaction pathways. This approach captures situated conditional combinations, clarifies causal asymmetry, and expands the theoretical understanding of user-context interactions.

Empirically, several configuration patterns stand out. Among the satisfaction pathways, Rule 6 (Safety = 5 and Cost Control Prioritization = 5; 45.37% coverage) stands out as the most representative configuration, showing that when safety and cost control are both at high levels, users report the most positive experiences. This finding confirms prior research emphasizing the central role of safety and economic affordability in shaping elderly users' satisfaction (63–67). However, our analysis goes beyond existing conclusions by revealing that high safety typically does not operate in isolation but must be configured with other enabling conditions to achieve high satisfaction. Rule 7 (Safety = 5 and Intergenerational Digital Inclusion = 3 and Demand for Inclusive Policies = 5; 11.11% coverage) highlights a different pathway, where high safety combined with policy and digital inclusion also leads to satisfaction, showing that institutional and social factors can complement core conditions. Rules 8 (Usefulness = 4 and Cultural Adaptability = 5; 17.59% coverage) and 9 (Usefulness = 4 and Health Management Needs = 4; 20.37% coverage) illustrate configurations centered on usefulness. This aligns with previous studies highlighting the importance of usefulness (65–67), but our findings add nuance by showing that improving usefulness alone is insufficient; it must be paired with cultural or health-related attributes to form a strong satisfaction pathway. Finally, Rule 10 (Intergenerational Digital Inclusion = 5 and Demand for Inclusive Policies = 3; 13.89% coverage) suggests that social and policy support can, in some contexts, independently foster satisfaction, despite its relatively low coverage.

In contrast, the dissatisfaction pathways (Rules 1–5) reflect distinct but concentrated negative mechanisms. Rule 1 (Safety = 2 and Usefulness = 2; 35.71% coverage) shows that low safety and usefulness significantly increase dissatisfaction, reaffirming their decisive role as fundamental conditions. Rule 2 (Economic Affordability = 3 and Cost Control Prioritization = 2; 35.71% coverage) highlights the sensitivity of economic dimensions. Rules 3 (Smart Technology = 1 and Economic Affordability = 1; 14.29% coverage) and 4 (Safety = 1 and Smart Technology = 1; 14.29% coverage) show how weak technological conditions combined with low safety or affordability exacerbate dissatisfaction. Rule 5 (Usefulness = 1 and Health Management Needs = 1; 14.29% coverage) further illustrates that poor usefulness and unmet health needs lead to dissatisfaction. Compared with the satisfaction pathways, these negative configurations are more straightforward, often triggered by the absence of basic conditions rather than complex synergies.

Taken together, safety and usefulness play decisive roles in both positive and negative configurations. High levels of these conditions interact with others to promote satisfaction, whereas low levels alone can trigger dissatisfaction. This asymmetry indicates that the mechanisms underlying satisfaction and dissatisfaction differ: satisfaction depends on multiple favorable conditions, while dissatisfaction often stems from basic deficiencies. This conclusion not only confirms previous research highlighting the importance of safety and usefulness (63, 65–67) but also extends it by revealing the multi-dimensional configurational logic required to achieve high satisfaction. This aligns with the core proposition of configurational theory and asymmetry theory (68).

### 5.2 Practical implications

Based on the configurational analysis, several targeted practical recommendations can be derived from specific rules.

First, according to Rule 6 (Safety = 5 and Cost Control Prioritization = 5), the combination of high safety and strong cost control is the dominant pathway to satisfaction. Therefore, ensuring device safety and reliability should be a top priority in product design and management. This includes improving protective structures, reducing operational risks, and implementing cost-control strategies to alleviate the economic burden on elderly users. For governments and healthcare institutions, measures such as price subsidies, simplified reimbursement procedures, or promoting affordable yet safe devices can further strengthen this pathway.

Second, Rules 8 and 9 (Usefulness = 4 and Cultural Adaptability = 5 / Health Management Needs = 4) highlight that combining usefulness with user needs adaptation constitutes another major satisfaction pathway. Practically, this means increasing the usefulness of treatment devices in older communities by installing them in neighborhood clinics, nursing homes, and rehabilitation centers. Moreover, device interfaces and functions should be tailored to the linguistic habits, cultural cognition, and health management needs of elderly users to reduce usage barriers and encourage active engagement.

Third, Rules 7 and 10 (involving Intergenerational Digital Inclusion and Demand for Inclusive Policies) emphasize the role of social and institutional support in fostering satisfaction. Policymakers and community managers can enhance these conditions by promoting digital inclusion programs (e.g., digital literacy training, simplified user interfaces) and improving public health policies (e.g., targeted subsidies, strengthened community service networks), thereby allowing these external factors to complement core conditions and expand the diversity of satisfaction pathways.

The dissatisfaction pathways (Rules 1–5) also offer clear directions for intervention. Rule 1 (Safety = 2 and usefulness = 2) indicates that dissatisfaction is most likely when both safety and usefulness are lacking, making this a top priority for policy and design action. Minimum safety standards and basic usefulness must be ensured to avoid this negative configuration. Rule 2 (Economic Affordability = 3 and Cost Control Prioritization = 2) suggests that insufficient cost control can exacerbate economic pressure for low-income elderly groups, highlighting the need for financial support measures such as subsidies or tiered pricing. Rules 3–5 (involving Smart Technology and Health Management Needs) show that technological complexity and mismatches with user needs can also trigger dissatisfaction. Therefore, when introducing smart technologies, designers should avoid excessive complexity and ensure compatibility with elderly users' abilities and health needs.

Overall, these practical insights derived from configurational pathways suggest that improving elderly users' experiences with treatment devices requires coordinated action across three levels: (a) basic assurance: by prioritizing safety and usefulness; (b) synergistic configuration: by aligning economic, cultural, and technological factors with user characteristics; and (c) social support: by leveraging digital inclusion and policy measures to expand satisfaction pathways.

## 6 Conclusion

With the acceleration of global population aging, elderly individuals in sub-health and pre-frailty states face increasing health risks that undermine quality of life. Home treatment devices provide a promising response by enabling accessible and adaptable care at home. Yet it remains unclear which antecedent conditions are most salient under broader environmental perspectives, and how these conditions interact configurationally to shape satisfaction.

To address these questions, this study first developed a systematic evaluation framework through literature review and expert refinement using FDM, covering product attributes, user needs, environmental constraints, and policy orientations. We then employed RSA to examine how these conditions interact in different configurations, revealing the inherent complexity and multiple pathways that jointly shape user satisfaction.

Our findings, grounded in configurational and asymmetry theories, show that satisfaction arises from joint configurations. For example, satisfaction arises when safety and usefulness work in combination with economic, cultural, and policy supports. By contrast, dissatisfaction is typically triggered by deficiencies in fundamental conditions. This extends traditional linear, symmetric approaches (e.g., SEM, AHP) by capturing nonlinear interdependencies, and complements methods such as KANO, DEMATEL, and fsQCA by foregrounding context-specific configurations using ordinal data without distributional assumptions. The rule-based configurational perspective proposed here provides a systematic theoretical account of multidimensional user-context interactions in elderly treatment device adoption.

Practically, this study offers targeted guidance for improving service systems and product design for elderly treatment devices. At the basic level, safety and usefulness should be treated as non-negotiables to avoid dissatisfaction pathways; in practice this often means reinforcing stability in hand-contact areas and providing unambiguous operation feedback so that common errors are prevented rather than corrected. At the configurational level, the RSA findings suggest aligning affordability, cultural adaptability, and health-management needs with policy support and digital-inclusion measures. In design terms, this translates into options that remain affordable across income segments, interfaces that reflect local language conventions and familiar symbols, and unobtrusive monitoring functions that support everyday self-management without adding cognitive load. Equally important is process: multidisciplinary teams (e.g., industrial designers, healthcare professionals, sociologists, and community managers) should co-develop requirements and iteratively test prototypes in community settings, so that clinical reliability, ergonomic comfort, and emotional reassurance are addressed together rather than sequentially.

Although this study has made preliminary progress in the demand assessment and configuration optimization of home treatment devices, there are still research gaps that need to be addressed. One limitation is that the analysis focused on home treatment devices as a broad category and primarily examined their relationship with overall user satisfaction, rather than evaluating specific types of devices or linking them directly to therapeutic outcomes. Future studies should refine this perspective by targeting particular devices and examining their performance in relation to health improvements. In addition, future research could expand the sample size and cover a broader range of regions and populations to further validate the universality and stability of this framework. At the same time, as technology advances and the needs of elderly populations evolve, the diversification and intelligence of device functions will further increase the complexity of demand configuration. Therefore, how to dynamically update demand and configuration models, in conjunction with artificial intelligence and big data technologies, will be a key focus of future research. Moreover, the literature review adopted a snowballing search rather than a full systematic review, which limits transparency and replicability. Future work could conduct a systematic or scoping review (e.g., PRISMA-guided) to build a more exhaustive indicator pool prior to expert evaluation. Finally, the interview and survey samples were drawn from an urban area in China, whereas the majority of the elderly population actually resides in rural regions. Rural older adults often live in different physical and social environments and may exhibit distinct patterns of technology acceptance compared to their urban counterparts. Future research should therefore incorporate rural elderly populations to examine potential urban–rural disparities in living contexts, health management needs, and smart device adoption, thereby enhancing the representativeness and generalizability of the findings.

## Data Availability

The raw data supporting the conclusions of this article will be made available by the authors, without undue reservation.
